# Mortality and revision risk after femoral neck fracture: comparison of internal fixation for undisplaced fracture with arthroplasty for displaced fracture: a population-based study from Danish National Registries

**DOI:** 10.1080/17453674.2020.1850940

**Published:** 2020-11-24

**Authors:** Bjarke Viberg, Trine Frøslev, Søren Overgaard, Alma Becic Pedersen

**Affiliations:** a Department of Orthopaedic Surgery and Traumatology, Lillebaelt Hospital – University Hospital of Southern Denmark, Kolding;; b Department of Regional Health Research, University of Southern Denmark, Odense;; c Department of Clinical Epidemiology, Aarhus University Hospital;; d Orthopaedics Research Unit, Department of Orthopaedic Surgery and Traumatology, Odense University Hospital, Odense;; e Department of Clinical Research, University of Southern Denmark, Odense, Denmark

## Abstract

Background and purpose — Hemiarthroplasty has lower reoperation frequency and better mobilization compared with internal fixation (IF) in patients with undisplaced femoral neck fractures (FNF), which might translate into lower mortality. In this population-based cohort study we compare the risk of mortality and reoperation in undisplaced FNF treated with IF and displaced FNF treated with arthroplasty in patients older than 70 years old. We assume that, per se, there is no difference in mortality risk between patients with a displaced and an undisplaced FNF.

Patients and methods — Hip fracture patients were identified in the Danish Multidisciplinary Hip Fracture Registry during 2005–2015. Data on medication, comorbidities, reoperation, and mortality were retrieved from other Danish medical databases. IF and arthroplasty patients were compared with regards to mortality and reoperation up to 5 years postoperatively. We calculated hazard ratios (HR) with 95% confidence intervals (CI) adjusting for relevant confounders.

Results — We included 19,260 FNF treated with arthroplasty and 10,337 FNF with IF. There was an increased risk of mortality for arthroplasty within 30 days, HR 1.3 (95% CI 1.3–1.4), compared with IF but not after 1 and 5 years. Arthroplasty patients had adjusted HRs for reoperation of 0.8 (0.8–0.9) within 1 year, 0.8 (0.7–0.9) within 2 years, and 0.8 (0.8–0.9) within 5 years postoperatively compared with IF.

Interpretation — Patients treated for a displaced FNF with arthroplasty had a higher risk of 30-day mortality compared with patients who had an undisplaced FNF treated with IF. It has to be considered that there were baseline differences in the groups but there was no difference in mortality risk up to 5 years post-surgery. Concerning reoperation, patients with a displaced FNF treated with arthroplasty had a lower risk of reoperation compared with IF for undisplaced FNF.

The general consensus on treating an undisplaced femoral neck fracture (FNF) with internal fixation (IF) (Dansk Sygeplejeråd et al. 2008, National Institute for Health and Care Excellence 2011, updated 2017) has been questioned by a recent meta-analysis demonstrating that treatment with hemiarthroplasty may reduce the relative risk of reoperation by 70% when compared with IF (Richards et al. 2020). The meta-analysis included 2 randomized clinical trials (RCTs) that demonstrated a 5% reoperation frequency in the hemiarthroplasty group compared with 20–21% in the IF group (Lu et al. 2017, Dolatowski et al. 2019).

There may be a lower reoperation rate but neither of the 2 RCTs (Lu et al. 2017, Dolatowski et al. 2019) found a difference in patient-reported outcome after 1 year. Dolatowski et al. (2019) did find a faster mobility (Timed-Up-And-Go) in the hemiarthroplasty (HA) group and better mobilization is also found when comparing arthroplasty with IF in displaced FNF (Gjertsen et al. 2008, Jiang et al. 2015). Better mobilization after hip fracture is important as it is associated with reduced mortality after surgery (Kristensen et al. 2016). Therefore, an arthroplasty for the undisplaced FNF may also reduce the mortality compared with IF. However, the current 2 RCTs were not large enough to address the mortality question. A difference in mortality could be investigated by assuming that there is no difference per se in mortality risk between patients with a displaced and undisplaced FNF. This assumption is supported by studies comparing IF for undisplaced FNF with arthroplasty for displaced FNF showing no difference in mortality (Mukka et al. 2020, Richards et al. 2020).

We therefore compared the mortality and reoperation after treatment of patients operated on by IF for undisplaced FNF compared with arthroplasty for a displaced FNF in patients ≥ 70 years old.

## Patients and method

### Study design

This is a population-based cohort study on patients with a FNF during 2005–2015 (both years included) and reported to the Danish Multidisciplinary Hip Fracture Registry. The age cutoff of 70 years was used since the Danish guideline recommends arthroplasty for patients with a displaced FNF who are older than 70 years as well as surgery for all hip fracture patients (Dansk Sygeplejeråd et al. 2008). Reporting is performed according to the RECORD extension to the STROBE guidelines (Benchimol et al. 2015).

### Setting

Denmark has approximately 5.8 million inhabitants and every Danish citizen is at birth issued a 10-digit Civil Personal Register number. This number allows unambiguous linkage between all Danish medical databases, and every person can therefore be traced until death or emigration. All Danish citizens are guaranteed free healthcare for any hospital treatment through the Danish National Health Service, which is why all patients will be treated in Denmark (Schmidt et al. 2014).

### Data sources

The Danish Multidisciplinary Hip Fracture Registry is a population-based clinical-quality database. Collecting data and reporting is mandatory for all hospital units treating hip fracture patients. A number of preoperative and perioperative data are prospectively collected including data on quality of inpatient care (Mainz et al. 2004). Data from the Danish Multidisciplinary Hip Fracture Registry is collected from the Danish National Patient Registry for Charlson comorbidity index (CCI) and reoperations (Dansk Tvaerfagligt Register for Hoftenaere Lårbensbrud 2017) and holds data on all hospital contacts including data on all surgical procedure dates and codes according to the Nordic Medico-Statistical Committee classification (Nordic Medico-Statistical Committee 2012). It is not mandatory to report BMI, which may explain reduced completeness. The completeness of the Danish National Patient Registry is considered to be 99.7% (Schmidt et al. 2015) and the positive predictive value of the hip fracture diagnosis is as high as 98% (Nymark et al. 2006, Hudson et al. 2013). Data from the Danish Multidisciplinary Hip Fracture Registry was also linked to the Danish Civil Registration System for vital status and migration for the entire Danish population (Schmidt et al. 2014). The Danish National Health Service Prescription Database has information on all prescriptions for reimbursed drugs dispensed by community pharmacies in Denmark and is recorded according to the Anatomical Therapeutic Chemical classification system (Johannesdottir et al. 2012).

### Study population

We used the Danish Multidisciplinary Hip Fracture Registry to identify the study population. All patients admitted to a hospital in Denmark with a hip fracture diagnosis code (ICD-10 DS720, DS721, DS722), surgical procedure code, and laterality were included. Using procedure codes (Nordic Medico-Statistical Committee 2012) the patients were categorized into an internal fixation or arthroplasty group. Internal fixation was defined as screw fixation or sliding hip screw and arthroplasty as hemiarthroplasty or THA (Table 1, see Supplementary data). If patient had bilateral hip fracture, only the first hip fracture was included in the study population. In the Danish Multidisciplinary Hip Fracture Registry, a code for undisplaced and displaced fracture exists. However, not all patients have the code and it has not been validated. Our national guidelines recommend arthroplasty for all displaced FNF patients above 70 years and IF for all undisplaced FNF. All patients above 70 years old treated with IF were therefore deemed to have an undisplaced FNF and those with arthroplasty as a displaced FNF.

### Outcome

Mortality was registered by date after surgery and collected from the Danish Civil Registration System. The follow-up for all patients was from surgery date to a maximum of 5 years or until end of follow-up.

Reoperation was defined as any open procedure: deep infection, change of implant, open reduction, or operation due to a new (i.e., periprosthetic) fracture (Tables 2 and 3, see Supplementary data).

### Variables

A priori, we identified potential confounders including age, sex, CCI, BMI, and medication. Age, sex, and BMI were retrieved from the Danish Multidisciplinary Hip Fracture Registry. From the Danish National Patient Registry (Schmidt et al. 2015) we retrieved information on comorbidity measured by CCI (Charlson et al. 1987) using discharge diagnoses up to 10 years prior to hip fracture operation. Both primary and secondary diagnoses, as well as diagnoses of inpatient and outpatient visits, were included. From the Danish National Health Service Prescription Database (Johannesdottir et al. 2012) data on several prescription medicine was retrieved that the author group a priori defined as potential confounders. NSAID, glucocorticoids, opioid, and antibiotics data was retrieved using prescriptions reimbursed within 90 days before operation. Data for antihypertensives, antidepressants, statins, and anticoagulants was retrieved using prescriptions reimbursed within 365 days before operation due to the larger packages of prescribed medicine.

Age was categorized as 65–74, 75–84, and ≥ 85 years old. Using the WHO classification, patients were categorized as underweight if BMI was < 18.5, normal weight if BMI was ≤ 18.5–24.9, overweight if BMI was 25–29.9, and obese if BMI was ≥ 30 (WHO 2000). 3 comorbidity levels were defined using the CCI score: 0 (none), 1–2 (low), 3 or more (high). Medication use was categorized using dichotomous values (yes/no) at baseline into NSAIDs, antihypertensives, glucocorticoids, antidepressants, statins, anticoagulants, opioids, and antibiotics.

### Bias

In Denmark, the commonly used approach to the hip is posterior; only 1 hospital uses the anterolateral approach routinely for patients with FNF. The posterior approach is associated with higher reoperation frequency compared with the lateral approach (van der Sijp et al. 2018) thereby possibly diminishing a difference in reoperation frequency between arthroplasty and IF.

### Study size

The 1-year mortality was the primary outcome, which for hip fracture patients in Denmark is approximately 27% (Danish Multidisciplinary Registry for Hip Fracture 2019). We estimated a 2% difference between the 2 groups. A 2-sample proportion sample-size calculation was therefore performed using respectively 26% mortality in the arthroplasty group and 28% mortality in the IF group. This yielded a sample size of 7,734 in each group, using 0.05 for alpha and 0.80 for power.

### Data access, linkage, and cleaning methods

The authors had complete access to data from the Danish Multidisciplinary Registry for Hip Fractures, the Danish National Patient Registry, the Danish National Database of Reimbursed Prescriptions, and the Danish Civil Registration System. This study draws on individual-level record linkage of data from nationwide medical registries using the unique Civil Personal Register number. The study was approved by the Danish Data Protection Agency (journal number 1-16-02-467-15) and the Danish Patient Safety Authority (case number 3-3013-1389/1).

### Statistics

The study population was divided into patients with an undisplaced FNF treated with IF and patients with a displaced FNF treated with an arthroplasty. We describe the study population according to the distribution of patients’ characteristics, tabulating the number and percentage of patients.

We used the Kaplan–Meier method to compute the mortality risk after surgery. Crude and adjusted Cox proportional hazards models were used to assess the choice of surgery impact on subsequent mortality up to 5 years after surgery. We adjusted for age at time of surgery, sex, BMI, comorbidity level, and medication, inclusive of NSAIDs, corticosteroids, antidepressants, opioids, and for reoperation the latter was included in the model as time-dependent variable.

In the survival analyses of reoperation, patients were followed from the date of surgery to reoperation, death or end of study period. We plotted cumulative incidence curves for reoperation for the 2 groups, using death as a competing risk. Crude and adjusted proportional sub-distribution hazards models accounting for competing risk of death (Fine and Gray 1999) were tested for assessing the choice of surgery impact on subsequent reoperation risk for different time intervals after surgery. They were not different from the Cox proportional hazards models, which were therefore applied instead. The HRs for reoperation were adjusted for age at time of surgery, sex, BMI, comorbidity level, and medication, inclusive of NSAIDs, corticosteroids, antidepressants, and opioids. All hazard ratios (HR) were calculated with 95% confidence intervals (CI). All data management and analyses were conducted in SAS 9.4 (SAS Institute, Cary, NC, USA).

### Ethics, funding, and potential conflicts of interest

This study has no direct implications for FNF patients. No funding was received for this study. There are no conflicts of interest related to this study. BV has, outside the study, received payment for lectures from Zimmer Biomet and Osmedic Swemac. SO has received a grant for research from Zimmer Biomet. AP and TF have no disclosures to declare.

## Results

**Figure 1.**
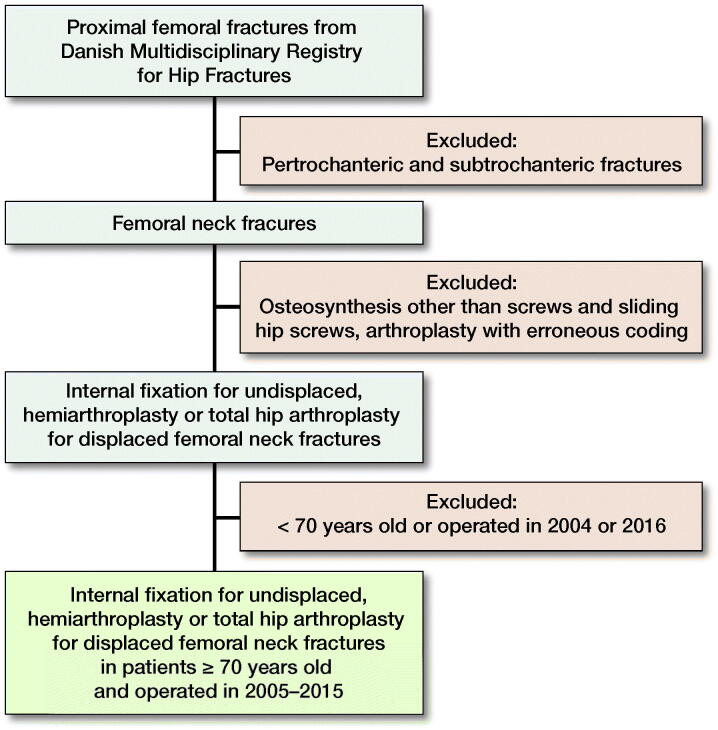
Flowchart of workup from the Danish Multidisciplinary Registry for Hip Fractures to study population of patients above 70 years treated with IF for undisplaced FNF and arthroplasty for displaced FNF.

During 2005–2015, there were 80,760 hip fracture operations in Denmark, of which 29,597 were in patients with an FNF over 70 years old treated with IF or arthroplasty (Figure 1). There were 10,337 patients with undisplaced FNF treated with IF and 19,260 displaced FNF treated with arthroplasty. The patient group with undisplaced FNF treated with IF were slightly younger, contained more males, and had a higher comorbidity level compared with the arthroplasty group (Table 4). The arthroplasty group consisted of 16,437 hemiarthroplasties and 2,823 THA. Patients receiving THA were younger and had a lower comorbidity level compared with hemiarthroplasty patients (Table 5).

**Table 4.** Demographic data of the study population and divided by type of osteosynthesis. Values are count (%)

Factor	Overall study population n = 29,597	Undisplaced FNF – IF n = 10,337	Displaced FNF – arthroplasty n = 19,260
Age at surgery			
70–74	3,861 (13)	1,639 (16)	2,222 (12)
75–84	12,969 (44)	4,487 (43)	8,482 (44)
≥ 85	12,767 (43)	4,211 (41)	8,556 (44)
Female sex	21,601 (73)	7,226 (70	14,375 (75)
Charlson Comorbidity Index			
0, none	11,901 (40)	3,998 (39)	7,903 (41)
1–2, low	12,061 (41)	4,280 (41)	7,781 (40)
≥ 3, high	5,635 (19)	2,059 (20)	3,576 (19)
BMI			
Missing	6,066 (21)	2,231 (22)	3,835 (20)
< 18.5	2,321 (7.8)	911 (8.8)	1,410 (7.3)
18.5–24	13,734 (46)	4,712 (46)	9,022 (47)
25–29	5,999 (20)	1,980 (19)	4,019 (21)
≥ 30	1,477 (5.0)	503 (4.9)	974 (5.1)
Medication			
NSAID	2,993 (10)	1,091 (11)	1,902 (9.9)
Antihypertensives	20,878 (71)	7,025 (68)	13,853 (72)
Glucocorticoids	1,252 (4.2)	443 (4.3)	809 (4.2)
Antidepressants	8,437 (29)	3,030 (29)	5,407 (28)
Statins	5,451 (18)	1,730 (17)	3,721 (19)
Anticoagulants	14,716 (50)	5,077 (49)	9,639 (50)
Opioids	2,168 (7.3)	737 (7.1)	1,431 (7.4)
Antibiotics	7,148 (24)	2,499 (24)	4,649 (24)

FNF, femoral neck fracture.

IF, internal fixation.

**Table 5.** Demographic data: patients with arthroplasty divided into hemiarthroplasty and total hip arthroplasty. Values are count (%)

	Hemiarthroplasty	Total hip arthroplasty
Factor	n = 16,437	n = 2,823
Age at surgery		
70–74	1,597 (9.7	625 (22)
75–84	7,174 (44)	1,308 (46)
≥ 85	7,666 (47)	890 (32)
Female sex	12,343 (75)	2,032 (72)
Charlson Comorbidity Index		
0, none	6,596 (40)	1,307 (46)
1–2, low	6,735 (41)	1,046 (37)
≥ 3, high	3,106 (19)	470 (17)
BMI		
Missing	3,425 (21)	410 (15)
< 18.5	1,206 (7.3)	204 (7.2)
18.5–24	4,712 (46)	9,022 (49)
25–29	7,656 (47)	1,366 (48)
≥ 30	3,340 (20)	679 (24)
Medication		
NSAID	1,592 (9.7)	310 (11)
Antihypertensives	11,866 (72)	1,987 (70)
Glucocorticoids	676 (4.1)	133 (4.7)
Antidepressants	5,446 (33)	840 (30)
Statins	4,164 (25)	798 (28)
Anticoagulants	8,315 (51)	1,324 (47)
Opioids	737 (7.1)	1,431 (7.4)
Antibiotics	3,984 (24)	665 (24)

### Mortality

**Figure 2.**
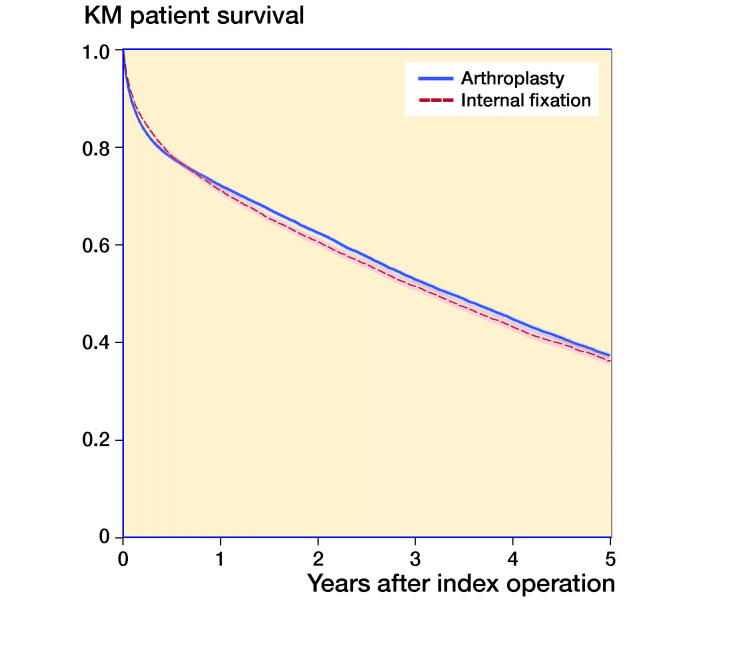
Kaplan–Meier survival plot with 95% CI comparing internal fixation for undisplaced fracture and arthroplasty for displaced fracture.

Within 30 days after surgery, the mortality was 11% in the arthroplasty group and 8.8% in the IF group. This corresponds to an adjusted HR of 1.3 (CI 1.2–1.4) for arthroplasty, 1.3 (1.2–1.4) for hemiarthroplasty while it was 1.0 (0.9–1.2) for THA when compared with IF (Figure 2 and Table 6).

**Table 6.** Adjusted regression analysis for mortality concerning internal fixation (IF) for undisplaced FNF versus arthroplasty, hemiarthroplasty and total hip arthroplasty (THA) for displaced FNF

Mortality Type of surgery	Deaths n	time, years	Person Mortality risk (%)	Adjusted HR (95% CI) ^a^	
Day 0–30					
IF		908	805	8.8	Reference
Arthroplasty	2,017	1,480	11	1.26 (1.17–1.37)	
Hemiarthroplasty	1,817	1,259	11	1.29 (1.19–1.40)	
THA	200	222	7.1	1.04 (0.89–1.22)Year 0–1	
IF		2,980	8,264	29	Reference
Arthroplasty	5,354	15,304	28	1.00 (0.96–1.05)	
Hemiarthroplasty	4,773	12,907	29	1.02 (0.98–1.07)	
THA	581	2,397	21	0.85 (0.77–0.93)	
Year 0–5					
IF		6,249	27,787	61	Reference
Arthroplasty	11,089	50,981	58	0.98 (0.95–1.01)	
Hemiarthroplasty	9,764	42,541	59	1.00 (0.97–1.03)	
THA	1,325	8,439	47	0.85 (0.80–0.90)	

CI, confidence interval. FNF, femoral neck fracture, HR, hazard ratio.

**^a^**Hazard ratios adjusted for age, sex, BMI, reoperation as a time dependent variable, comorbidity level, and medication, inclusive of NSAIDs, corticosteroids, antidepressants, opioids.

The mortality within 1 year was 28% in the arthroplasty group and 29% in the IF group. This corresponds to an adjusted HR of 1.0 (1.0–1.1) within 1 year for arthroplasty, 1.0 (1.0–1.1) for hemiarthroplasty, while it was 0.9 (0.8–0.9) for THA when compared with IF (Figure 2 and Table 6).

### Reoperation

**Figure 3.**
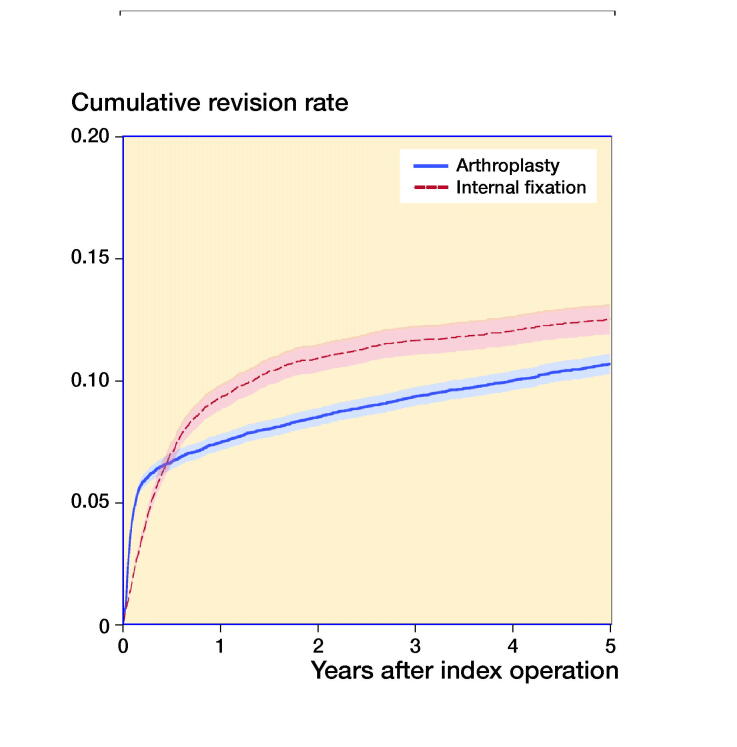
Cumulative incidence of reoperation for any reason over time of internal fixation for undisplaced fracture and arthroplasty for displaced fracture with 95% CI.

Within 1 year after surgery, the reoperation rate was 7.5% in the arthroplasty group and 9.3% in the IF group. This corresponds to an adjusted HR of 0.8 (0.8–0.9) when comparing arthroplasty with IF (Figure 3 and Table 7). Both hemiarthroplasty and THA had a lower reoperation risk than IF with similar results even after 2 and 5 years (Table 7).

**Table 7.** Adjusted regression analysis for reoperation concerning internal fixation (IF) for undisplaced FNF versus arthroplasty, hemiarthroplasty and total hip arthroplasty (THA) for displaced FNF

Follow-up Type of surgery	Cumulative incidence (%)	Adjusted HR (95% CI) ^a^
Year 0–1		
IF	9.3	Reference
Arthroplasty	7.5	0.82 (0.75–0.89)
Hemiarthroplasty	7.9	0.87 (0.80–0.95)
THA	5.0	0.52 (0.44–0.62)
Year 0–2		
IF	11	Reference
Arthroplasty	8.5	0.79 (0.73–0.85)
Hemiarthroplasty	8.9	0.84 (0.77–0.90)
THA	6.2	0.55 (0.46–0.64)
Year 0–5		
IF	13	Reference
Arthroplasty	11	0.85 (0.79–0.91)
Hemiarthroplasty	11	0.88 (0.82–0.95)
THA	8.9	0.64 (0.56–0.74)

CI, confidence interval. FNF, femoral neck fracture,

HR, hazard ratio.

**a** Hazard ratios adjusted for age, sex, BMI, comorbidity level, and medication, inclusive of NSAIDs, corticosteroids, antidepressants, opioids.

## Discussion

We assumed that there is no difference per se in mortality risk between patients with a displaced and undisplaced FNF. Patients receiving an arthroplasty for displaced FNF had a higher mortality after 30 days (11% vs. 8.8%) but not after 1 and 5 years compared with patients treated with IF for undisplaced FNF. However, patients with arthroplasty had, after 1 year, a 7.5% reoperation frequency compared with 9.3% in the IF group.

The higher risk of 30-day mortality in the arthroplasty group may be due to selection bias. We see a baseline difference with higher age, more males, and higher comorbidity level in the hemiarthroplasty group compared with IF. All these factors are associated with higher mortality, thereby influencing the 30-day result. We also see a substantially lower age in the smaller THA group (15% of the arthroplasties), thereby demonstrating that THA is performed in primarily healthier patients. Another factor concerning the baseline differences could be bone cement implantation syndrome when using an arthroplasty, leading to increased perioperative mortality (Middleton et al. 2014). A possible confounder could be the degree of posterior tilt (posterior angulation of the femoral head in comparison with the femoral neck) introduced in 2009 (Palm et al. 2009). If an undisplaced FNF has more than 20 degrees’ posterior tilt, then the risk of reoperation when using IF is increased. This could lead to surgical bias, as a surgeon might be more prone to choose an IF for a 72-year-old patient with a 20-degree posterior tilt compared with an 82-year-old patient. This could explain some of the baseline difference, which, however, could also be due to an underlying confounding in the population sustaining a displaced or undisplaced FNF, but previous studies have not found a major difference between groups (Mukka et al. 2020, Richards et al. 2020). There is, however, no difference in the 1-year mortality between hemiarthroplasty and IF despite the baseline difference. This could be due to better mobilization, less pain, and lower reoperation frequency in patients treated with hemi­arthroplasty.

We found a 1-year reoperation frequency of 7.5% in the arthroplasty group compared with 9.3% in the IF group. The arthroplasty reoperation frequency is nearly double compared with the Norwegian and Swedish registers (Rikshöft 2017, Norwegian Hip Fracture Register 2018). This is probably due to the exclusive use in Denmark of the posterior approach, which is associated with higher reoperation frequency compared with the lateral and anterolateral approach (van der Sijp et al. 2018) that is primarily used in Norway and Sweden (Rikshöft 2017, Norwegian Hip Fracture Register 2018). By shifting to a different approach in Denmark the reoperation frequency could be lowered (Sköldenberg et al. 2010) and thereby perhaps mortality too.

There has been 1 similar study published but it focuses on reoperation frequency and compares hemiarthroplasty with IF (Gjertsen et al. 2011). It demonstrates a reoperation frequency of 11% for IF in undisplaced FNF, which is higher in this study, but they included all reoperations including simple removal of implant, which we excluded. They report only 3% reoperation for hemiarthroplasty for displaced FNF, which is much lower than our 7.5% but could be due to the surgical approach. That study also demonstrated that hemiarthroplasty had the lowest degree of pain, and that patients were most satisfied and reported the highest quality of life. Our sub-analysis comparing hemiarthroplasty with THA confirms this, as the 1-year mortality is 29% in the hemiarthroplasty group and 21% in the THA group. By not including THA, selection bias occurs when assessing mortality as some of the healthiest FNF patients receive a THA. This is an important aspect in our study. The RCT by Dolotowski et al. (2019) demonstrated a lower 24-month mortality in the hemiarthroplasty group but was not sufficiently powered to show a statistically significant difference. An ongoing Swedish RCT will give us more knowledge on mortality as it has calculated sample size on a composite variable of reoperation and mortality (Wolf et al. 2020).

Concerning limitations there are, as discussed, baseline differences that may be due to the earlier explanations but there could also be unmeasured confounding or confounding by indication. We did not use the code for displaced/undisplaced fracture due to missing data and lack of validation, thereby not knowing the number of patients with a displaced FNF who were treated with IF and undisplaced FNF with arthroplasty. In addition, we did not measure the radiographs for displaced/undisplaced, posterior tilt, and implant positioning, which could all have an impact on the results. However, we do not believe that these measures would have a great impact as the study uses the results of everyday practice. We do not have functional or patient-reported outcomes that potentially could demonstrate a difference. The study has a major strength in using national registries with complete follow-up that can follow patients on an individual level across the country. We have included several possible biases for adjustment such as comorbidity, medication, and BMI. Another strength is the large cohort, thereby enabling the possibility to demonstrate small differences.

In conclusion, patients treated for a displaced FNF with arthroplasty had a higher risk of 30-day mortality compared with patients who had an undisplaced FNF treated with IF. It has to be considered that there were baseline differences in the groups but there was no difference in mortality risk up to 5 years post-surgery. Concerning reoperation, patients with a displaced FNF treated with arthroplasty had a lower risk of reoperation compared with IF for undisplaced FNF.

## Supplementary data

Tables 1–3 are available as supplementary data in the online version of this article, http://dx.doi.org/10.1080/17453674.2020.1850940
